# Supplementary material for: Patterns of Intron Gain and Loss in Fungi
Source: PLoS Biol. 2004 Nov 30;2(12):e422. doi: 10.1371/journal.pbio.0020422 (PMC532390; doi:10.1371/journal.pbio.0020422)
Supplement: Table S1 — Also available at http://genes.mit.edu/NielsenEtAl/. (4.3 MB ZIP). [file pbio.0020422.st001.zip › NielsenEtAl/html/714.html]

AN0870.1.NCU05390.1.MG02370.1.FG01591.1


```
 CLUSTAL W (1.82) Multiple Sequence Alignments - Introns Inserted


Sequence 1: AN0870.1	314 aa
Sequence 2: MG02370.1	323 aa
Sequence 3: NCU05390.1	319 aa
Sequence 4: FG01591.1	291 aa
Alignment Length: 335 aa
Number Identitical Residues: 188 aa
Alignment Score (without introns) 8434


MG02370.1 	------MAAIKKEAPVASPT----GLSLYSKFALSGAVCCSVTHGMVTPLDV2VKTRIQL
NCU05390.1	MAPSTPKVDAVVEKAKAAAAPEMSAASLYGRFALAGAICCSVTHGALTPVDV2VKTRIQL
FG01591.1 	----------MKQQP-----------TKHKRTALT-------EKQATNNIYS~VKTRIQL
AN0870.1  	-------MAAKIDAPAAAPAPQLSGLQLYSRFAFAGAVCCSITHGALTPVDV2VKTRIQL
          	            : . ::.:.. :.   : : *::.: ..:  :   . :   *******

MG02370.1 	DPQTYNRGMIGGFRQVIQNEGAGALLTGLGPTVAGYFLQGAFKFGGYEFFKAQSINTLGL
NCU05390.1	DPVTYNNGMIGGFKKVVQNEGAGALLTGVGPTFAGYFLQGAFKFGGYEFFKQQSINLLGL
FG01591.1 	DPATYNRGLIGGFRQVVKNEGAGALLTGVGPTFAGYFLQGALKFGGYEFFKQQWIDALGY
AN0870.1  	DPVTYNRGMIGGFRQVIANEGAGALLTGFGPTAAGYFLQGAFKFGGYEFFKQQWINQLGY
          	** ***.*:****::*: **********.*** ********:********* * *: ** 

MG02370.1 	ETASKYRTSVYLASAAAAEFFADIALCPLEATRIRLVSDPSYANGLIGGFTKMLRTEGVG
NCU05390.1	ETATNNRTAVYLASSAAAEFFADIALCPLEATRIRLVSQPTYASGLISGFGKMLKNEGIG
FG01591.1 	ETASKNRTAVYLASSATAEFFADIALCPLEATRIRLVSEPTYASGLVSGFGKIVKNEGFG
AN0870.1  	ESASKNRTAIYLASSATAEFFADIALCPLEATRIRLVSQPEFASGLLSGFSKILKNEGVG
          	*:*:: **::****:*:*********************:* :*.**:.** *:::.**.*

MG02370.1 	AFYAGFGPILFKH2LHLAAQHVLTLFLR~VPYTMTKFVVYEKVAEAIFRVYPKESLSDGM
NCU05390.1	AFYAGFGPILFK-~-------------Q2IPYTMSKFVVYEKVAEAVYSVYPKKDMSDSA
FG01591.1 	ALYAGFGPILFK-~-------------Q2IPYTMAKFVVFEKVSEAVFRTFPKKDLSDGM
AN0870.1  	AFYSGFGPILFK-~-------------Q2VPYTMAKFVAFEKFSEAIYGVVDKNTLSDGG
          	*:*:********               : :****:***.:**.:**:: .  *: :**. 

MG02370.1 	QTVVNLGSGLMAGFAAAIVSQPADTMLSKINK~TKGLPGEGTTSRLIKIAKELGLRGSFG
NCU05390.1	QTAVNLGSGLIAGFAAAIVSQPADTMLSKINK0TPGAPGESTTSRLVKIAKELGLKGSYT
FG01591.1 	QTVANLGSGLIAGFAAAIVSQPADTMLSKINK~TQGLPGEGTVSRLVKIGKELGIRGSYS
AN0870.1  	KTVVNLGSGLLAGFAAAIVSQPADTMLSKINK~TQGLPGEGTTTRLIKIAKELGLKGSFS
          	:*..******:********************* * * ***.*.:**:**.****::**: 

MG02370.1 	GIGARLFM~VGTLTAGQFAIYG~DLKK~ALGATGGVEIAK--
NCU05390.1	GIGARLFM~VGTLTAFQFAIYG1DVKK~SLGATSGVELAK--
FG01591.1 	GIGARLFM~VGTLTAGQFAIYG~DLKK0AMGATGGVEIAA--
AN0870.1  	GIGARLFM0IGTITAGQFAIYG~DIKR~LLGATGGVEIAPSK
          	******** :**:** ****** *:*:  :***.***:* :.
```
